# Acute Transverse Myelitis (ATM):Clinical Review of 43 Patients With COVID-19-Associated ATM and 3 Post-Vaccination ATM Serious Adverse Events With the ChAdOx1 nCoV-19 Vaccine (AZD1222)

**DOI:** 10.3389/fimmu.2021.653786

**Published:** 2021-04-26

**Authors:** Gustavo C. Román, Fernando Gracia, Antonio Torres, Alexis Palacios, Karla Gracia, Diógenes Harris

**Affiliations:** ^1^ Department of Neurology, Neurological Institute, Houston Methodist Hospital, Houston, TX, United States; ^2^ Weill Cornell College of Medicine, Cornell University, New York, NY, United States; ^3^ Department of Neurology, Texas A&M University College of Medicine, Bryan, TX, United States; ^4^ Neurology Service, Hospital Paitilla, Panama City, Panama; ^5^ Faculty of Health Sciences, Interamerican University of Panama, Panama City, Panama; ^6^ Neurology Service, Hospital Santo Tomás, Panama City, Panama; ^7^ Infectious Disease Service, Hospital Santo Tomás, Panama City, Panama; ^8^ Neuroradiology Service, Complejo Hospitalario Metropolitano, CSS (Caja de Seguro Social), Panama City, Panama; ^9^ Interamerican University of Panama, Panama City, Panama; ^10^ Neurosurgery Service, Complejo Hospitalario Metropolitano, CSS, Panama City, Panama

**Keywords:** COVID-19, neurological complications, SARS-CoV-2 neurotropism, myelitis, transverse myelitis, COVID-19 ChAdOx1 nCoV-19 vaccine

## Abstract

**Introduction:**

Although acute transverse myelitis (ATM) is a rare neurological condition (1.34-4.6 cases per million/year) COVID-19-associated ATM cases have occurred during the pandemic.

**Case-finding methods:**

We report a patient from Panama with SARS-CoV-2 infection complicated by ATM and present a comprehensive clinical review of 43 patients with COVID-19-associated ATM from 21 countries published from March 2020 to January 2021. In addition, 3 cases of ATM were reported as serious adverse events during the clinical trials of the COVID-19 vaccine ChAdOx1 nCoV-19 (AZD1222).

**Results:**

All patients had typical features of ATM with acute onset of paralysis, sensory level and sphincter deficits due to spinal cord lesions demonstrated by imaging. There were 23 males (53%) and 20 females (47%) ranging from ages 21- to 73- years-old (mean age, 49 years), with two peaks at 29 and 58 years, excluding 3 pediatric cases. The main clinical manifestations were quadriplegia (58%) and paraplegia (42%). MRI reports were available in 40 patients; localized ATM lesions affected ≤3 cord segments (12 cases, 30%) at cervical (5 cases) and thoracic cord levels (7 cases); 28 cases (70%) had longitudinally-extensive ATM (LEATM) involving ≥4 spinal cord segments (cervicothoracic in 18 cases and thoracolumbar-sacral in 10 patients). Acute disseminated encephalomyelitis (ADEM) occurred in 8 patients, mainly women (67%) ranging from 27- to 64-years-old. Three ATM patients also had blindness from myeloneuritis optica (MNO) and two more also had acute motor axonal neuropathy (AMAN).

**Conclusions:**

We found ATM to be an unexpectedly frequent neurological complication of COVID-19. Most cases (68%) had a latency of 10 days to 6 weeks that may indicate post-infectious neurological complications mediated by the host’s response to the virus. In 32% a brief latency (15 hours to 5 days) suggested a direct neurotropic effect of SARS-CoV-2. The occurrence of 3 reported ATM adverse effects among 11,636 participants in the AZD1222 vaccine trials is extremely high considering a worldwide incidence of 0.5/million COVID-19-associated ATM cases found in this report. The pathogenesis of ATM remains unknown, but it is conceivable that SARS-CoV-2 antigens –perhaps also present in the AZD1222 COVID-19 vaccine or its chimpanzee adenovirus adjuvant– may induce immune mechanisms leading to the myelitis.

## Introduction

Neurological complications of coronavirus disease 2019 (COVID-19) are well recognized (1–3) and affect both the central nervous system (CNS) and the peripheral nervous system (PNS). Neurological injury results from the affinity of the COVID-19 etiological agent, the Severe Acute Respiratory Syndrome coronavirus 2 (SARS-CoV-2), for the angiotensin-converting enzyme 2 (ACE2) receptor present in neurons and glial cells endowing high neuroinvasive potential to SARS-CoV-2 compared to previous coronaviruses. The high frequency of anosmia during the acute infection probably reflects viral invasion of the olfactory bulbs. Cells with abundant ACE2 receptors are infected first by this coronavirus including nasal epithelium cells, ciliated bronchial epithelial cells and type II pneumocytes, explaining the severity of the pulmonary involvement. Also, the presence of ACE2 receptors for the viral S protein in endothelial cells correlates with the frequent vascular complications of COVID-19 resulting from endotheliitis and microvascular brain injury (4) that induces the host’s immune response with cytokine storm, hyperinflammation, coagulopathy, thrombosis and embolism resulting in ischemic and hemorrhagic strokes and multisystemic complications affecting lungs, heart, kidneys and liver.

According to Borchers and Gershwin (5), ATM is a rare neurological condition in adults with an estimated incidence ranging between 1.34 and 4.6 cases per million annually with a mean age of 35-40 years. We report a patient with SARS-CoV-2 infection in Panama who developed acute transverse myelitis (ATM) and we present the results of a comprehensive review of COVID-19-associated myelitis that yielded 42 additional cases reported in 21 countries worldwide (6–44) published from March 2020 to January 2021 during year 1 of the pandemic (**Table 1** and **Supplementary Table 1A**). Furthermore, 3 ATM serious adverse events were reported with the ChAdOx1 nCoV-19 (AZD1222) vaccine trials (45, 46).

**Table 1:** Summary of SARS-CoV-2-Associated Myelitis Published Cased from March-2020 until January-2021.

Case	Country	Sex/Age years	Myelitis Type		Lesion Level				Other Clinical Features	Ref.
			ATM	LEATM	C	CT	Th	Conus		
1	CN	M/66	ATM				Th10			Zhao (6)
2	IR	M/60		LEATM	C1-4					Saberi (7)
3	GB	M/40	ATM		C1-2				ADEM Brainstem rhombencephalitis	Wong (8)
4	IT	W/54		LEATM		C2-Th6			Bulbomedullary lesions	Zanin (9)
5	DK	W/28		LEATM	X	X	X	X	Medulla oblongata to conus medullaris	Sarma (10)
6	ES	W/69		LEATM		C7 - Th1			Medulla to C7- Th1	Sotoca (11)
7	DE	M/60	ATM				Th9		Late lesions Th3-5 Th9-10	Munz (12)
8	IT	W/64	ATM				Th8		ADEM – NMO Monoclonal gammopathy CSF SARS-CoV-2 (+)	Novi (13)
9	IT	W/22	ATM		X					Giorgianni (14)
10	US	W/61		LEATM		C1-Th1			AMAN	Valiuddin (15) Maideniuc (16)
11	AE	M/32		LEATM		C2-Th-L				Al Ketbi (17)
12	BR	W/42	ATM		C5				Trigeminal nucleus	Barros- Domingues (18)
13	IR	M/21		LEATM		C1-Th				Zoghi (19)
14	US	M/24		LEATM			Th7-12			Durrani (20)
15	CH	M/63	ATM				Th10			Zachariadis (21)
16	TR	M/48	ATM		C2-3				ADEM CSF: SARS- CoV-2 (+)	Otluoglu (22)
17	QA	M/52		LEATM			Th3-10			Abdelhady (23)
18	US	M/44		LEATM		C5-7 Th3-6		conus medullaris	ADEM	Utukuri (24)
19	US	W/40	ATM		C1				ADEM Pons, medulla	McCuddy (25)
20	AU	M/60		LEATM			Th7-10			Chow (26)
21	US	G/3		LEATM		C1 to Th6			Lower medulla to Th6	Kaur (27)
22	MD	M/27		LEATM		C4-Th5			HIV (+)	Lisnic (28)
23	IN	W/59	ATM				Th6-7			Chakraborty (29)
24	IR	M/63		LEATM		C7-Th12				Hazrati (30)
25	BR	W/51		LETM			Th6-10		Lumbar radiculitis	Corrêa (31)
26	IT	W/70	ATM			C7-Th1			AMAN Anti-GD1b IgM	Masuccio (32)
27	IR	W/53	ATM				Th8-10			Baghbanian (33)
28	IT	M/64	ATM							Rifino (34)
29	IT	M/64	ATM							Rifino (34)
30	ES	M/50	ATM		C5-C6					Águila- Gordo (35)
31	TR	G/14		LEATM	C2-5					Güler (36)
32	MX	M/73		LEATM	C1 - C3-6				Atlas to C3-C6 cervical spondylotic myelopathy	Guadarrama - Ortiz (37)
33	US	M/26		LEATM		C4-7 Th5-8			Edema optic nerves MOG-IgG-mediated NMOSD	Zhou (38)
34	ID	W/45	ATM				Th3-4			Munir (39)
35	GB	W/33		LEATM		C1-7 Th2			ADEM Brain & pontomedullary	Paterson (40)
36	GB	W/27	ATM					Conus medullaris	ADEM diffuse T2 white matter and corticospinal lesions	Paterson (40)
37	GB	M/48		LEATM			Th5-6 Th10-11	Conus medullaris		Paterson (40)
38	IR	M/47		LEATM		C2-Th2				Advani (41)
39	IR	W/67		LEATM	C3-C6					Advani (41)
40	PK	M/56		LEATM			Th4-Th8			Ali (42)
41	IR	G/11		LEATM			Th3-Th6			Nejad- Biglari (43)
42	BE	W/38		LETM		C3-Th4				Fumery (44)
43	PA	M/72		LEATM		C2-Th9				Román-Gracia (this report)

Names of Countries: Australia = AU, Belgium = BE, Brazil = BR, China = CN, Denmark = DK, Germany = DE, India = IN, Indonesia = ID, Iran = IR, Italy = IT, Mexico = MX, Moldova = MD, Panama = PA, Pakistan = PK, Qatar = QA, Spain = ES, Switzerland = CH, Turkey = TR, United Arab Emirates = AE, United Kingdom = GB, United States of America = US.

ADEM, acute disseminated encephalomyelitis; AMAN, acute motor axonal neuropathy; ANA, antinuclear antibodies; AQP4, aquaporin-4; ATM, acute transverse myelitis; (C, cervical; CSF, cerebrospinal fluid; G, girl; HIV, human immunodeficiency virus; LEATM, Iongitudinally-extensive acute transverse myelitis; M, man; MOG-IgG, myelin oligodendrocyte glycoprotein antibody-immune globulin G; NMOSD, neuromyelitis optica spectrum disorder; SARS-CoV-2, severe acute respiratory syndrome-coronavirus-2, Th, thoracic; W, woman.

## Case Description

A previously-healthy 72-year-old man presented to the emergency department at a hospital in Panama City, Panama, complaining of sudden difficulty to urinate. The urologist diagnosed neurogenic bladder and placed a Foley catheter. Three days later the patient developed dysesthesias in arms and legs and weakness of all four limbs. Neurologic examination showed 3+/5 strength in the upper extremities and 1+/5 in the lower limbs with spastic paraplegia, generalized hyperreflexia, bilateral Babinski, and spontaneous pyramidal jerking of both legs; sensory examination disclosed decreased proprioception in the legs and a tactile sensory level below Th_9_. The patient was alert and oriented; higher cortical functions, cranial nerves and cerebellar examination were all intact.

He denied fever, headache, ageusia, anosmia, fatigue, diarrhea or upper respiratory symptoms during the past 3 weeks. Past medical history was negative except for hypertension controlled with enalapril. The SARS-CoV-2 RNA PCR nasal swab test was negative on 2 occasions. His wife was asymptomatic, but her nasal swab test was positive, and she had SARS-CoV-2 antibodies. The patient’s serology demonstrated recent infection with SARS-CoV-2 IgG index = 3.53 (normal <1.6) and IgM index = 5.1 (normal <0.6). Chest X-rays showed mild cardiomegaly but no evidence of consolidation or pleural effusion. Chest computerized tomography (CT) scan was normal. Electrocardiogram showed mild left ventricular hypertrophy. The patient was afebrile and his general physical evaluation was normal. Respiratory rate 16 breaths per minute, oxygen saturation 98% on room air, blood pressure 130/80 mmHg, heart rate 78 beats per minute and temperature 36.8°C.

Laboratory results showed normal white blood cell count (8,100/μL) with normal hemoglobin (14.7g/dl). Inflammatory markers showed elevated C-reactive protein at 1.7mg/dl and high erythrocyte sedimentation rate at 51mm/hr (normal range 0-10mm/hr). Coagulation profile was normal. Protein C, Protein S, Antithrombin III, and activated Protein C resistance were within normal limits. Hematology consult found no associated pathology. Hepatic and thyroid function tests were normal. VDRL and HIV tests were negative. Autoimmune immunological screening was negative for lupus anticoagulant, anti-nuclear antibodies (ANA), anti-neutrophil cytoplasmic antibodies, rheumatoid factor, anti-cardiolipin, and complement C3, C4. Rheumatology consult found no underlying disease. Aquaporin-4 antibody (anti-AQP4) and myelin oligodendrocyte glycoprotein antibody IgG (anti-MOG-IgG) in serum were both negative (Quest diagnostics Nichols Institute, CA).

**Figure 1:**
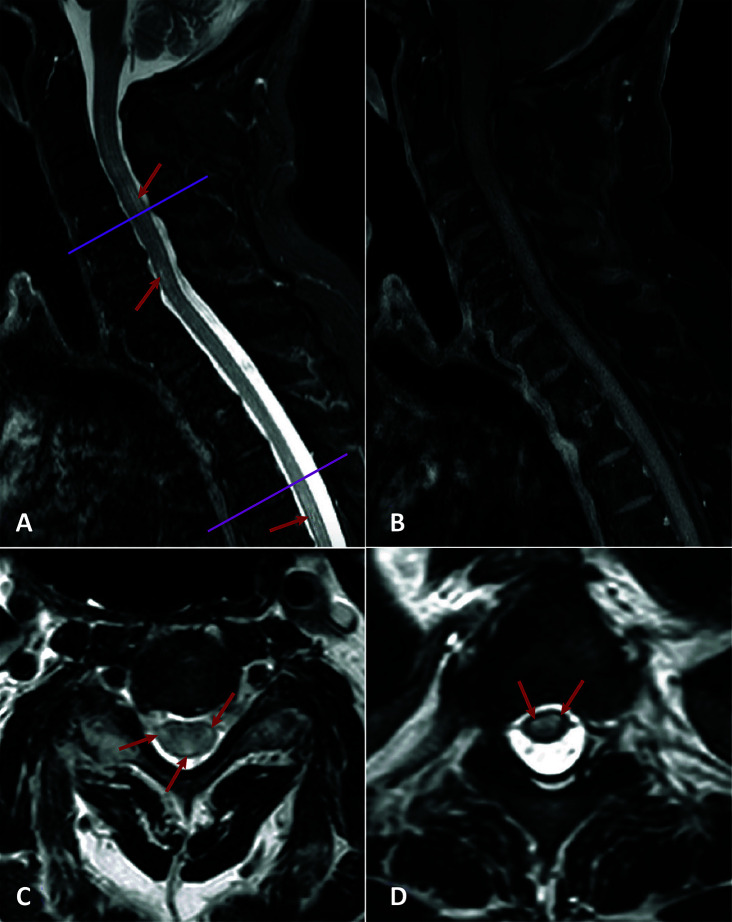
Spinal cord MRI. **(A)** Sagittal Short-T1 Inversion Recovery: Mild cervical cord thickening and diffuse hyperintensities in cervical and dorsal cord (red arrows). **(B)** Sagittal T1 + gadolinium. No contrast enhancement. **(C, D)** Axial T2 (C4-5 and T3-4 levels) showing diffuse cord hyperintensities (red arrows).

Gadolinium-enhanced magnetic resonance imaging (MRI) of the brain was normal. MRI of the spinal cord revealed mild cervical and thoracic cord enlargement and swelling with diffuse hyperintensities. On the axial projections, cord hyperintensities at C_4_-C_5_ and Th_3_-Th_4_ were observed with irregular patchy imaging but without contrast enhancement consistent with ATM (**Figure 1**). No apparent hemorrhagic components were present, and the conus medullaris had normal appearance.

Cerebrospinal fluid (CSF) showed no cells, hyperproteinorraquia of 76mg/dl and normal glucose. Meningitis CSF panel was negative for bacteria, yeast and viruses. Gram, acid-fast bacilli and fungus stains were negative. CSF oligoclonal bands (IgG) demonstrated 3 well-defined gamma restriction bands that were not present in serum (Quest diagnostics Nichols Institute, CA).

The patient was treated with a pulse dose of IV methylprednisolone 1g/d for 5 days, enoxaparin 40 mg daily, followed by IV gamma-globulin (IVIG) 30g/day for five days. Oral prednisone was prescribed for the next 30 days. He recovered partial strength in his upper limbs (4+/5) but the severe spastic paraplegia (1+/5) and the neurogenic bladder remained unchanged. He is undergoing physical therapy and rehabilitation treatment.

## Case-Finding Review

We performed a comprehensive search of the literature using PubMed, Medline, Scopus, Web of Science, EMBASE, and Google Scholar up to January 5, 2021. For PubMed we used the following key search terms: (“Myelitis, Transverse” [MeSH] OR “Myelitis” [All Fields] OR “Myelitis, Acute” [All Fields] OR “Encephalomyelitis” [All Fields] OR “Neuromyelitis Optica” [MeSH] OR “Myeloneuropathy” [All Fields] OR “Encephalomyelitis, Acute Disseminated” [MeSH] OR “Acute Disseminated Encephalomyelitis” [All Fields]) AND (“COVID-19” [MeSH Term] OR “SARS-CoV-2” [MeSH Term] OR “coronavirus” [All Fields]). **Table 1** and **Supplementary Table 1A** list the total 43 patients reported in 21 countries worldwide, as follows: 7 cases from Iran (IR), 6 each from Italy (IT) and the United States of America (US), 4 from the United Kingdom (GB), 2 cases each from Brazil (BR), Spain (ES), and Turkey (TR), plus single case reports from Australia (AU), Belgium (BE), China (CN), Denmark (DK), Germany (DE), India (IN), Indonesia (ID), Mexico (MX), Moldova (MD), Panama (PA), Pakistan (PK), Qatar (QA), Switzerland (CH), and the United Arab Emirates (AE). Patient 10 from the US was published twice (15, 16).

## Results

Early reports of neurological complications of COVID-19 from China (2) and France (3) included no cases of ATM. Therefore, it was unexpected to collect 43 cases of COVID-19-associated myelitis in a period of 10 months around the world. Given a total of 86 million COVID-19 cases as of 5 January 2021 (coronavirus.jhu.edu) the incidence of myelitis is 0.5 per million. Based on a single hospital COVID-19 series with 1760 patients from Italy (34), SARS-CoV-2-associated ATM may represent 1.2% of all neurological complications of COVID-19.

COVID-19-associated ATM was reported in 23 males (53%) and 20 females (47%) ranging in age from 21 to 73 years (mean age 49 years) excluding children. There were three age groups: (i) *Pediatric cases:* Patient 21, a 3-year-old Navajo girl in the USA (27), Patient 31, a 14-year-old girl in Turkey (36), and Patient 41, an 11-year-old girl from Iran (43). (ii) *Young adult cases:* 13 patients, 7 men and 6 women, ages 21-42 years with mean age of 29 years. (iii): *Older adults:* 27 patients, 18 men and 9 women, ranging from 44-73 years (mean age 58 years).

The main manifestations of the spinal cord lesions based on clinical examination included two major groups, quadriplegia and paraplegia. There were 27/40 patients (58%) with tetraparesis/quadriplegia resulting from cervical cord-upper thoracic cord lesions compared with 15/40 (42%) with acute paraparesis/paraplegia from thoracic cord lesions. The anatomical distribution of the spinal cord lesions by MRI imaging was reported in 40 cases (**Table 1** and **Supplementary Table 1A**). Localized ATM lesions affected ≤3 cord segments in 12 cases (30%) at cervical (5 cases) and thoracic cord (7 cases) levels, and 28 cases (70%) had longitudinally-extensive ATM (LEATM) involving ≥4 spinal cord segments in cervicothoracic (18 cases) and thoracolumbar-sacral regions (10 cases). In 3 case reports the lesions are described as ‘myelitis’ without further clinical information. Cervical cord lesions extended in some cases to the brainstem causing rhombencephalitis (8), as well as involvement of pons and medulla oblongata. Patient 4 from Denmark (10) had the most extensive lesions reported affecting the entire spinal cord from the medulla oblongata to the conus medullaris. Patients 18, 36 and 37 had lesions affecting the conus medullaris.

The latency period from the onset of COVID-19 symptoms to the first neurological manifestations followed a dual distribution: (i) *Short latency:* 15 hours to 5 days in 11/34 patients (32%) and (ii) *Long latency:* 10 days to 6 weeks in 68% (**Table 1** and **Supplementary Table 1A**). The shorter latency period may indicate a direct neurotropic effect of SARS-CoV-2 during the initial infection causing para-infectious myelitis. Longer latency periods may indicate a post-infectious neurological complication resulting from the host response to the virus. No particular geographic origin, distribution by sex or age group, nor clinical picture were associated with shorter or longer latency periods. Treatments included steroids in most patients, along with IVIg in a few instances; some patients also received respiratory support. There were 2 deaths reported (Patient 17 from Qatar and Patient 23 from India). SARS-CoV-2 RNA PCR in the CSF was positive in 2 cases (18, 24).

## ATM and Other Neuroinflammatory Syndromes

According to Hartung and Aktas (47) a number of neuroimmunological disorders affecting CNS and PNS are expected to occur during COVID-19. Although the immune mechanisms causing ATM remain unknown other neurological disorders of neuroimmune nature were reported concurrently with the myelitis (**Table 1** and **Supplementary Table 1A**). These included acute disseminated encephalomyelitis (ADEM), neuromyelitis optica (NMO) and acute motor axonal neuropathy (AMAN).

## Acute Disseminated Encephalomyelitis (ADEM)

Reichard et al. (48) reported the neuropathological findings, extensive vascular lesions and perivenous demyelination of ADEM in association with COVID-19. Myelitis as part of ADEM was diagnosed in 8/40 patients (20%) summarized in **Table 1** and **Supplementary Table 1A**. In contrast with the overall male preponderance in this series, ADEM with ATM affected predominantly women (67%) ranging in age from 27-64 years (mean age 43 years). Lesions revealed by spinal cord MRI included LEATM from medulla oblongata and cervico-thoracic cord (Th_6_) in Patient 3 (8); cervicothoracic spinal cord lesions down to the conus medullaris in Patient 18 (24); C_1-7_-Th_2_ in Patient 35 (40); and, Th_5-6_ and Th_10-11_ down to the conus medullaris in Patient 37 (40). ATM at Th_8_ level occurred in Patient 8 (13); at C_2-3_ level in Patient 16 (22); pons and medulla-cord junction in Patient 19 (25); and, intramedullary lesion of the conus medullaris in Patient 36 (40). Brain MRI lesions consistent with ADEM included among others, multiple T1 post-Gd enhancing white matter lesions plus bilateral edema of the optic nerves; hyperintense FLAIR lesions in the medial temporal lobe; bilateral lesions involving cerebral white matter, corpus callosum and brainstem including pons and medulla-cord junction.

## Neuromyelitis Optica (NMO) and NMO Spectrum Disorders (NMOSD)

NMO and NMOSD are relatively common conditions in neuroimmunology (49–51) previously reported after SARS-CoV-2 infection (52). We found 3 patients with myelitis and visual loss due to optic nerve edema diagnosed with NMO. Patient 8 (13), is a 64-year-old woman with ATM and visual loss. Patient 33 (38), is a 26-year-old Hispanic man from the USA with positive MOG-IgG antibodies who developed papilledema, blindness and dysesthesias of the upper extremities due to bilateral optic neuritis and LEATM cord lesions from C_4_-Th_2_. Patient 25, from Rio de Janeiro, Brazil (31) is a 51-year-old Caucasian woman, with a 2-week history of COVID-19 who developed band-like dysesthesias at the Th_6-10_ dermatomes, urinary retention, leg numbness and paraparesis. Brain MRI showed enhancing T2/FLAIR lesions in anterior fornix and subfornical organ. Spinal cord MRI demonstrated LEATM at Th6-10 with lumbar radiculitis. Serum ANA was positive (1:320). Anti-AQP4 antibodies were positive in serum and CSF. This encephalomyeloradiculitis is probably a form of COVID-19-associated NMOSD. According to Jarius et al. (53) NMO and NMOSD are caused in >80% of cases by pathogenetic IgG autoantibodies to AQP4 but only 1 case was positive in this cohort. A 15-year-old Caucasian boy with SARS-CoV-2-associated NMO reported by de Ruijter at al (52). had blindness without myelitis but with positive anti-MOG-IgG antibodies.

## Acute Motor Axonal Neuropathy (AMAN)

AMAN may be found in patients with clinical Guillain-Barré syndrome (GBS), an acute immune-mediated polyradiculoneuropathy reported as the most common form of peripheral nerve lesion in patients with COVID-19 (54, 55). Based on electrophysiological features GBS can be classified into several subtypes including acute inflammatory demyelinating polyneuropathy (AIDP), acute motor and sensory axonal neuropathy (AMSAN), and AMAN (54, 56).

We found reports of 2 patients that presented concurrently AMAN with ATM indicating simultaneous involvement of CNS and PNS. Patient 10, a 61-year-old woman from the US (15, 16) developed quadriparesis due to LEATM affecting C_1_-Th_1_ and concurrent AMAN with negative anti-MOG-IgG and anti-AQP4-IgG antibodies. Patient 26, a 70-year-old woman from Italy (32) developed quadriparesis from ATM at C_7_-Th_1_ and AMAN with positive anti-GD1b IgM antibodies.

## Other Immune/Inflammatory Factors

In addition to the above patients other immune or inflammatory mechanisms may have contributed to COVID-19-associated ATM. Patient 22 is a 27-year-old HIV-positive man from Moldova (28) who developed paraplegia due to LEATM involving C_4_-Th_5_. Patient 32 is a 72-year-old man from Mexico (37) with preexisting cervical spondylotic myelopathy that evolved to tetraparesis after SARS-CoV-2 infection due to ATM at the C_1_-C_3_-C_6_ levels.

## Discussion

The neurotropism of the coronaviruses in general and SARS-CoV-2 in particular has been well demonstrated (57–60). Moreover, the numerous neurological complications of COVID-19 are well recognized (1–4, 34, 40, 47, 48, 54, 55, 57, 61). Symptoms reflecting central nervous system involvement include headache, anosmia and dysgeusia, agitation, delirium, and impaired consciousness (1, 61). Stroke is common, probably reflecting the endoteliitis (62) and small-vessel brain lesions (4) causing brain hemorrhages, arterial and venous thromboses, and subarachnoid hemorrhage, as well as rare cases of acute hemorrhagic necrotizing encephalopathy (48, 61). Neuropathological examination of fatal adult cases of COVID-19 (63–65) showed in addition to vascular lesions (4) low-grade localized encephalitis affecting brainstem respiratory and cardiovascular centers (63).

According to Paterson et al. (40) the postulated mechanisms causing ATM and the various neurological syndromes associated with SARS-CoV-2 include, either individually or in combination, direct viral neuronal injury (57–60) and the host’s secondary hyperinflammation syndrome (61, 66, 67). SARS-CoV-2 enables interleukin (IL)-1 synthesis and release (68) leading to inflammasome activation. Also, IL-6, a proinflammatory mediator, is elevated in COVID-19 and induces CNS immune responses (68). Type I interferon (IFN) is dysregulated in COVID-19 and can affect innate and acquired immunity (69). COVID-19 patients exhibit increased circulating levels of IL-2, IL-8, IL-17, granulocyte colony-stimulating factor, granulocyte-macrophage colony-stimulating factor, interferon gamma-induced protein 10, and monocyte chemoattractant protein 1 (69, 70). IFN release can result in inflammation and immune system suppression (70). These immune factors may lead to the so-called “cytokine storm” syndrome that triggers coagulopathy and thrombosis (71). Also, of critical importance during COVID-19 are the para- and post-infectious inflammatory or immune-mediated neurological disorders (72–75), also observed after vaccination (76, 77), that affect both the CNS and the PNS causing GBS, ADEM, NMOSD, and ATM, among others.

## Acute Transverse Myelitis (ATM)

### Definition and Differential Diagnosis

The term ATM is used here to identify patients with myelitis described during COVID-19. Most COVID-19-associated ATM cases reported here fulfill the strict definition of the Transverse Myelitis Consortium Working Group (78) requiring clinical evidence of bilateral sensory, motor, or autonomic dysfunction referable to the spinal cord, and confirmed by MRI images.

ATM is different from acute flaccid myelitis or AFM (79) the predominantly pediatric form of acute flaccid paralysis with anterior myelitis or “polio-like syndrome” with spinal cord gray matter lesions. Epidemiological data for pediatric cases of AFM from a national surveillance program in the US (80) reported as of July 2020 a total of 633 cases of AFM with a median age of 5.3 years with peaks in 2014, 2016 and 2018. Non-polio enteroviruses, including EV-D68 and EV-A71, are the most frequent etiological agents (81). Children are relatively unaffected by SARS-CoV-2, probably because of low ACE2 receptors in the olfactory mucosa (82) and there has been no increase in cases of AFM up to July 2020. Patient 21 (27), a 3-year-old Navajo girl, is the youngest pediatric case of SARS-CoV-2-associated ATM reported in this series. Three weeks after an asymptomatic COVID-19 infection she developed a flaccid quadriparesis as a result of LEATM extending from the lower medulla and C_1_ to the Th_6_ spinal cord segments. These lesions are clearly different from those of pediatric AFM (83).

According to West and colleagues (84), ATM remains a rare immune-mediated neurological condition with an estimated incidence of up to 3 per 100,000 patient years (0.003%). ATM can be caused by autoimmune, inflammatory, and infectious agents but the main differential diagnosis is with multiple sclerosis. Clinical features and imaging usually eliminate from the differential diagnosis of ATM other noninflammatory conditions such as traumatic, compressive, neoplastic or vascular lesions (84).

### Pathogenesis of COVID-19-Associated ATM

The latency period between SARS-CoV-2 infection and onset of the neurological symptoms was unknown in many instances because of asymptomatic COVID-19. In most cases (68%) the latency period ranged from 10 days to 6 weeks; in the remaining 32% (11/34 cases) the latency period ranged from hours to 5 days; the shortest period was 15 hours for Patient 22, the HIV-positive man from Moldova (28). Very short latency periods of respectively 2 and 3 days occurred for Patient 11 from UAE (17) and Patient 17 from Qatar (23). It is unknown if these two patients had been previously infected with the Middle East Respiratory Syndrome coronavirus (MERS-CoV).

The neurological complications of viral infections can be either para-infectious, i.e., due to direct viral neurotropism, or post-infectious, i.e., resulting from immune-mediated reactions against the virus (48, 57–59, 61–68). Except for the three para-infectious cases mentioned above, most cases of SARS-CoV-2-associated myelitis had longer latency periods suggesting a post-infectious origin.

According to Blackburn and Wang (72), the proposed mechanisms of post-infectious neurological disorders include molecular mimicry, epitope spreading, bystander activation and polyclonal B-cell activation. Molecular mimicry is due to the presence in microorganisms of epitopes that share marked similarity in peptide sequence or three-dimensional structure to host’s antigens. Therefore, lymphocytes activated by the infection may cross-react with self-antigens. In epitope spreading the specific initial response to an antigen is broadened to include other different epitopes. Also, during the immune response to a highly virulent pathogen autoreactive lymphocytes may be activated during the inflammatory cascade resulting in autoimmunity by “bystander activation.” Finally, polyclonal B-cell activation may occur with chronic viral infections that persist in the host such as herpesviruses. Molecular mimicry and bystander activation appear to be the most likely mechanisms explaining SARS-CoV-2-associated ATM. The antibodies reported in patients with COVID-19-associated ATM included 3 cases of positive anti-MOG-IgG antibodies and single cases of positive anti-GD1b IgM antibodies, ANA, and anti-AQP4 antibodies in serum and CSF.

## Post-Vaccination ATM

Neurological complications of vaccination were first recognized in 1885 with Pasteur’s rabies vaccine obtained from rabbits’ spinal cords. More recently, in 1977, we reported 21 cases of GBS and brain demyelination (77) resulting from the use in Colombia of the suckling mouse brain (SMB) rabies vaccine containing neural tissue antigens causing neurological complications that included GBS, ADEM, chronic leukoencephalitis, and myelitis (77). Concurrent involvement of CNS and PNS occurs in post-viral infections such as Zika (75). These lesions resemble those of experimental autoimmune encephalomyelitis induced in animals with the use of myelin antigens and Freund’s adjuvant (74, 76, 84). Current rabies vaccine obtained from tissue culture of human diploid cells eliminated this problem.

The US national vaccination campaign in 1976 against the A New Jersey “swine flu” influenza using the A/NJ/1976/H1N1 vaccine was associated with increased incidence of GBS (85). Nachamkin et al. (86) postulated that *Campylobacter jejuni* antigens that mimic human gangliosides capable of inducing an anti-GM_1_ antibody response could have caused GBS. *Campylobacter* antigens were not present in any of the vaccines examined. However, these authors demonstrated that the 1976, 1991-1992, 2004-2005 influenza vaccines induced IgG and IgM anti-GM_1_ antibodies in mice. Recent cases of ATM following H1N1 vaccination have been reported (87–90) indicating that influenza vaccines may induce immune mechanisms targeting the spinal cord. It may be important to notice that the COVID-19 ChAdOx1 nCoV-1 vaccine (AZD1222) contains chimpanzee adenovirus antigens as adjuvant.

In 2003, an experimental vaccine (AN1792) containing synthetic aggregated Aβ42 fragments with QS-21 as adjuvant targeting the amyloid precursor protein with the aim of preventing the development of Alzheimer’s disease resulted in meningoencephalitis in 6% of the vaccinated patients (91). The trial was discontinued and neuropathology studies (92) confirmed the presence of meningoencephalitis with strong MCH class I immunoreactivity in collapsed amyloid plaques and multinucleated giant cells suggesting that a fragment of β-amyloid was the possible origin of the post-vaccination reaction.

Numerous other vaccines have caused neurological post-vaccination complications including diphtheria-tetanus-polio, measles, mumps, rubella, Japanese B encephalitis, and pertussis. According to Karussis and Petrou (76), the most recent cases of CNS demyelination after vaccination include vaccines against influenza, human papilloma virus, hepatitis A or B, measles, rubella, yellow fever, anthrax, meningococcus and tetanus. Other than GBS, ATM, and ADEM, other post-vaccination reactions include neuromyelitis optica (NMOSD), isolated ophthalmoplegia, brachial neuritis and other mononeuropathies. Post-vaccination reactions have declined with the use of recombinant proteins, rather than *in vivo* infected animal tissue (73). Recent research on the immunopathogenesis of ATM (93) has emphasized the role of interleukins IL-6 and IL-17. In myelitis, IL-6 is elevated in the CSF and predicts disability (94). Production of both IL-6 and IL-17 by peripheral blood mononuclear cells is increased in ATM (94). IL-17 regulates cytokines (TNFα, IL-1β and IL-6) to stimulate IL-6 production by astrocytes. The role of adjuvants as contributing factors to the immune and inflammatory reactions to vaccines has also been emphasized (95).

### ChAdOx1 nCoV-19 Vaccine (AZD1222) Trials

The ChAdOx1 nCoV-19 vaccine (AZD1222) consists of a replication-deficient chimpanzee adenoviral ChAdOx1 containing the SARS-CoV-2 structural surface vector glycoprotein antigen (spike protein; nCoV-19) gene (45). The safety and efficacy report of four randomized controlled trials conducted in Brazil, South Africa and Great Britain for the AZD1222 COVID-19 vaccine informed the occurrence of three cases of ATM as serious adverse events (45, 46).

One participant developed ATM 14 days after ChAdOx1 nCoV-19 booster vaccination and was diagnosed as idiopathic, short segment, spinal cord demyelination possibly related to vaccination (45, 46). The second participant developed ATM 10 days after a first vaccination with ChAdOx1 nCoV-19. It was initially assessed as possibly vaccine-related but later considered unlikely when further investigation revealed pre-existing, but previously unrecognised, multiple sclerosis (45, 46). The third patient with ATM occurred in a control subject 68 days after receiving the meningococcal conjugate (MenACWY) vaccine. Initially considered possibly related, it was finally considered unlikely to be vaccine-related by neurological experts (45, 46). However, no information was provided regarding COVID-19 infection in this unvaccinated subject. These three ATM serious adverse events resulted in temporarily halting of the vaccine trial until the affected participants began to show signs of recovery.

The occurrence of three reported ATM cases among 11,636 participating subjects is extremely high considering the worldwide incidence of 0.5/million COVID-19-associated ATM cases found in this report during year 1 of the pandemic. Moreover, Agmon-Levin et al. (96) in a systematic review (1970–2009) fund in 39 years only 37 reported cases of ATM in association with several different vaccines.

## Conclusion

This review confirms that ATM is not uncommon as a neurological complication associated with COVID-19 infection around the world, responsible perhaps for 1.2% of all neurological complications caused by this coronavirus. It occurs acutely in a small number of patients as a para-infectious manifestation but most cases of SARS-CoV-2-associated ATM have longer latency periods suggesting a post-infectious origin. These facts suggest probable viral antigen(s) in SARS-CoV-2 target the spinal cord –perhaps also present in the COVID-19 vaccine AZD1222 or its chimpanzee adenovirus adjuvant– and may induce immune mechanisms leading to ATM. Research to identify the responsible antigen(s) and the immunopathogenesis of COVID-19-associated ATM must be encouraged.

## Data Availability Statement

The datasets presented in this article are not readily available. Requests to access the datasets should be directed to gcroman@houstonmethodist.org.

## Author Contributions

GR and FG drafted the manuscript. FG, AT, AP, KG, and DH provided clinical data. GR, FG, and KG participated in the search of the literature. All authors contributed to the article and approved the submitted version.

## Funding 

Prof. Román’s research is funded by the Blanton Endowed Chair, the Wareing Family Research Fund and the David Cabello Research Fund at Houston Methodist Hospital.

## Conflict of Interest

The authors declare that the research was conducted in the absence of any commercial or financial relationships that could be construed as a potential conflict of interest.

## Supplementary Material

Supplementary Table 1A Clinical and MRI Data of Published Cases of SARS-CoV-2-Associated Myelitis (March-2020 January-2021).
