# Supplementary material for: Acute Transverse Myelitis (ATM):Clinical Review of 43 Patients With COVID-19-Associated ATM and 3 Post-Vaccination ATM Serious Adverse Events With the ChAdOx1 nCoV-19 Vaccine (AZD1222)
Source: Front Immunol. 2021 Apr 26;12:653786. doi: 10.3389/fimmu.2021.653786 (PMC8107358; doi:10.3389/fimmu.2021.653786)
Supplement: Supplementary Table 1A — Clinical and MRI Data of Published Cases of SARS-CoV-2-Associated Myelitis (March-2020 January-2021). [file Table_1.pdf]

Supplementary Table 1A. Clinical and MRI Data of Published Cases of SARS-CoV-2-Associated Myelitis (March-2020 January-2021)

#	Country	Sex/ Age years	COVID-19 Manifestations	L-P	Lesion level	Clinical Features	First Author [Ref]
1	China	M/66	Fever, fatigue, pneumonia	1 wk	ATM Th_{10}	Flaccid paraplegia, incontinence, T_{10} sensory level	Zhao (6)
2	Iran	M/60	Fever, N&V, pneumonia	2 wk	LEATM C_{1-4}	Paraparesis, incontinence	Saberi (7)
3	UK/Nigerian	M/40	Fever, dyspnea, malaise, cough, diarrhea, unsteady gait	10 d	ADEM Brainstem rhombencephalitis ATM C1-2	Diplopia, oscillopsia, limb ataxia, RUE numbness, hiccups, dribbling eating/drinking, facial and tongue weakness, upbeat nystagmus, ataxia of LE	Wong (8)
4	Italy	W/54	A/D, pneumonia	1 wk	LEATM Bulbomedulla C_2-Th_6 MRI brain DWL	LOC, seizures, Intubated on ventilator	Zanin (9)
5	Denmark	W/28	Fever, LBP, cough	1 wk	LEATM Medulla oblongata to conus medullaris	Dorsal column sensory loss below Th_5 incontinence	Sarma (10)
6	Spain	W/69	Fever, cough	1 wk	LEATM Medulla to C_7 Late central necrosis C_7- Th_1	Face/hand hypoesthesia, hyperreflexia, paraparesis, incontinence	Sotoca (11)

7	Germany	M/60	Pneumonia	1 wk	ATM Th_9<br>Late $Th_{3\text{-}5}$ $Th_{9\text{-}10}$	Paraparesis, incontinence, Babinski, Th_9 hypoesthesia	Munz (12)
8	Italy	W/64	Vitiligo, monoclonal gammopathy, flu symptoms, A/D	2wk	ADEM<br>Optic neuritis ATM Th_8<br>CSF: SARS- CoV-2 (+)	Blindness, RLE sensory deficit, Th sensory level hyperreflexia. L Babinski	Novi (13)
9	Italy	W/22	Fever, dyspnea, LOC, pneumonia	2 wk	No MRI Cervical cord	Flaccid tetraparesis, incontinence, Hyperreflexia	Giorgianni (14)
10	US	W/61	Rhinorrhea, chills, afebrile	1 wk	LEATM<br>C_1-Th_1<br>AMAN	Quadriparesis, hyporeflexia, incontinence, sensory level C_3	Valiuddin (15)<br>Maideniuc (16) duplicate publication
11	UAE	M/32	Fever, flu-like	2d	LEATM<br>C_2-Th-L	Paraparesis, incontinence	Al Ketbi (17)
12	Brazil	W/42	Mild URS, coryza	3wk	Trigeminal nucleus<br>ATM C_5	Paresthesias/ hypoesthesia LUE, left hemithorax, left hemiface	Barros-Domingues (18)
13	Iran	M/21	Fever, N&V, malaise, chills, cough	2 wk	LEATM C_1-Th<br>MRI brain DWL lesions	Paraparesis, incontinence. Drowsiness	Zoghi (19)
14	US	M/24	Fever, N&V, chills, pneumonia	2 wk	LEATM<br>$Th_{7\text{-}12}$	Flaccid paraplegia, incontinence	Durrani (20)
15	Switzerland	M/63	Fever, rhinorrhea, H/A, myalgia	2 wk	No MRI Thoracic myelitis Th_{10}	Paresthesias feet, incontinence, paraplegia	Zachariadis (21)
16	Turkey	M/48	H/A, anosmia, fatigue, myalgias, A/D	10 d	ADEM<br>ATM $C_{2\text{-}3}$<br>CSF: SARS- CoV-2 (+)	Headache, fatigue, myalgias	Otluoglu (22)
17	Qatar	M/52	Fever, abdominal pain, pneumonia	3d	LEATM Ventral cord $Th_{3\text{-}10}$	Flaccid paraparesis, incontinence, no sensory loss, died	Abdelhady (23)

18	US	M/44	Urinary retention, leg numbness, paraparesis	2d	ADEM LEATM C_{5-7} Th_{3-6} conus medullaris	Lethargy, dysarthria, ataxia UE, paraparesis, urinary retention	Utukuri (24)
19	US	W/40	Fever, cough, chest pain, SOB. Obesity, DM2, pregnancy 30 w gestation		ADEM Pons, medulla ATM	Intubated, paraplegia, hyperreflexia, weak UE, normal sensation	McCuddy (25)
20	Australia	M/60	Fever, A/D, cough	2 wk	LEATM Th_{7-10}	Paraparesis, hyperreflexia, incontinence, sensory loss Th_{10}	Chow (26)
21	US Navajo	G/3	Asymptomatic	3 wk	LEATM Lower medulla, C_1 to Th_6	Flaccid quadriparesis, sensory loss below neck, areflexia, incontinence, respirator	Kaur (27)
22	Moldova	M/27	Pneumonia	15 hrs	LEATM C_4-Th_5	HIV (+) Paraplegia, sensory loss	Lisnic (28)
23	India	W/59	Fever	4d	ATM Th_{6-7}	Flaccid paraplegia, areflexia, sensory loss below Th_{10}, died	Chakraborty (29)
24	Iran	M/63	Fever, flu-like	4d	LEATM C_7-Th_{12}	Flaccid paraplegia, incontinent, sensory loss below Th_8	Hazrati (30)
25	Brazil	W/51	Fever, cough, A/D, abdominal dysesthesia	1wk	LETM Th_{6-10} Lumbar radiculitis	Urinary retention, LLE weakness Anti-AQP4 ANA 1:320	Corrêa (31)
26	Italy	W/70	Fever, anosmia, myalgia	2 wk	ATM dorsal cord C_7-Th_1	Quadriparesis, hyperreflexia, Babinski,	Masuccio (32)

					AMAN<br>Anti-GD1b IgM	incontinence, sensory loss	
27	Iran	W/53	Pneumonia, LBP, urinary incontinence	2 wk	ATM<br>Th_8-Th_{10}	Flaccid paraplegia, areflexia, Babinski, incontinence, sensory loss Th_{10}-Th_{11}	Baghbanian (33)
28	Italy	M/64			Myelitis		Rifino (34)
29	Italy	M/64			Myelitis		Rifino (34)
30	Spain	M/50	Fever, cough, asthenia, LBP	4d	ATM C_5-C_6<br>Disk herniation	Dysesthesias lower limbs & genitals, incontinence, paraparesis	Águila- Gordo (35)
31	Turkey	G/14	R hemiplegia		LEATM $C_{2\text{-}5}$	R hemiplegia	Güler (36)
32	Mexico	M/73	Fever, quadriparesis, urinary retention, constipation, neck pain	6 wk	LEATM Atlas (C1) C3-C6 cervical spondylotic myelopathy	Quadriparesis, hyperreflexia, Babinski,ataxia, incontinent	Guadarrama - Ortiz (37)
33	US<br>Hispanic	M/26	Cough, visual loss OS>OD, eye pain, numbness, neck discomfort	1d	Edema optic nerves<br>LEATM<br>$C_{4\text{-}7}$ $Th_{5\text{-}8}$	Blindness, papilledema, MOG-IgG- mediated NMOSD	Zhou (38)
34	Indonesia	W/45	Shortness of breath	5d	No MRI<br>Th $_{3\text{-}4}$	Weakness and numbness of legs<br>Sensory level Th $_{3\text{-}4}$	Munir (39)
35	UK White	W/33	Fever	2d	ADEM Brain pontomedullary<br>LEATM $C_{1\text{-}7}Th_2$	Headache, confusion, deterioration in consciousness	Paterson (40)
36	UK Asian	W/27	Cough, fever, A/D	8d	ADEM diffuse T2 White matter and corticospinal lesions<br>Intramedullary ATM lesion conus medullaris	Sensory loss, inbalance, spastic gait, hypertonia, ankle clonus, pyramidal weakness, loss of pain-touch sensation in feet	Paterson (40)
37	UK White	M/48	Cough, fever, dyspnea	2 wk	LEATM $Th_{5\text{-}6}$ $Th_{10\text{-}11}$ down to conus medullaris	Numbness umbilical level; unsteady gait. Weak hip flexion; brisk reflexes; loss vibration. Sensory ataxia. Sensory level Th_{10}	Paterson (40)
38	Iran	M/47	Fever, cough, diarrhea	10 d	LEATM C_2-Th_2	Flaccid paraplegia, arreflexia, incontinent	Advani (41)

39	Iran	W/67	Loss of appetite	4wk	LEATM C_3-C_6	Spastic paraparesis	Advani (41)
40	Pakistan	M/56	Fever, fatigue	4d	LETM Th_4-Th_8	Spastic paraparesis, incontinence	Ali (42)
41	Iran	G/11	Fever, abdominal pain	3d	LETM Th_3-Th_6	Paraparesis urinary and stool incontinence	Nejad- Biglari (43)
42	Belgium	W/38	Cough, myalgia, dyspnea	2wk	LETM C_3-Th_4	Paraparesis, sensory loss below Th_4	Fumery (44)
43	Panama	M/72	Afebrile, urinary retention, dysesthesias, tetraparesis	3d	LEATM C_2-Th_9	Quadriparesis, hyperreflexia, Babinski, urinary & fecal retention, proprioceptive loss LE, sensory loss below Th_9	Román-Gracia (this report)

Abbreviations: A/D=anosmia/dysgeusia, ADEM=acute disseminated encephalomyelitis, AMAN=acute motor axonal neuropathy, ATM=acute transverse myelitis, C=cervical, d=days, DWL=diffuse white matter lesions, G=girl, H/A=headache, HIV=human immunodeficiency virus, hrs=hours, L=lumbar, LBP=low-back pain, LE=lower extremities, LEATM=longitudinally-extensive acute transverse myelitis, LOC=loss of consciousness, L-P=latency period, M=man, MOG- IgG=myelin oligodendrocyte glycoprotein antibody – immune globulin G, MRI=magnetic resonance imaging, NMOSD=neuromyelitis optica spectrum disorder, N&V=nausea and vomiting, OD=right eye, OS=left eye, Th=thoracic, UAE=United Arab Emirates, UE=upper extremities, UK=United Kingdom, US=United States of America, W=woman, wk=week
